# Portable infrared pupillometry in critical care

**DOI:** 10.1186/s13054-016-1349-7

**Published:** 2016-06-22

**Authors:** Merlin D. Larson, Vineeta Singh

**Affiliations:** Department of Anesthesia and Perioperative Care, University of California, San Francisco, CA 94143 USA; Department of Neurology, University of California, San Francisco, CA 94143 USA; Departments of Anesthesiology and Neurology, University of California, San Francisco, CA 94143-0648 USA

**Keywords:** Pupil, Pupillometry, Critical care, Brain injury

## Abstract

Infrared pupillometry was introduced in 1962 but portable instruments that use this technology have only recently become available in the hospital setting. Questions surrounding the accuracy of these instruments have been addressed by documenting the inter-observer agreement on pupillary measurements and also by comparisons with standard pen-light examinations. The following commentary summarizes the development of these devices and provides a wider perspective on how the pupil and its reflexes might be used in providing care for patients with critical illness.

## Background

Couret et al. [1] recently presented a large observational study comparing automated pupillometry with the standard subjective pupillometry in brain-injured patients. The study confirms previous reports [2, 3] of discordance between these two methods of examining pupillary size, anisocoria, and the pupillary light reflex (PLR). Importantly, the discovery of discrepancy in measurement in both directions as measured by bedside nurses highlights the lack of standardization of this essential examination. As such, there is potential for unnecessary interventions, delays in diagnosis, and adverse clinical outcomes.

When the technique of portable infrared pupillometry was introduced over 25 years ago [4], there was the concern that eye movements of the subject or hand movements of the examiner might introduce artifacts that would falsely mimic a PLR. Modern techniques of tracking the pupil have eliminated this concern. For example, movement of the pupillometer or the target while measuring fixed metal holes will result in scans that are flat because of algorithms embedded in the instrument that maintain a steady image of the aperture. Thus, the attending physician can be assured that changing trends of pupil size and PLR originate from the patient and not from subjective interpretations of the pen-light examination from different examiners, or from noise in the recording device. Confidence in the accuracy of sequential measurements may be a primary reason to use this new technology.

## Main text

Robert Whytt correctly argued with the influential physician Albrecht von Haller 250 years ago that the PLR required a pathway through the brain. Whytt could not have appreciated why his idea would be so important to physicians in the 21st century, who no longer define life in terms of cardiac function. Light on the retina is a benign non-noxious stimulus and the reflex it produces can be rapidly non-invasively recorded and time-stamped. It is not depressed by paralyzing agents that can confound the assessment of brain stem function by other methods (breathing, eye movements, corneal reflexes). An intact PLR reveals that the observed patient was alive at that time because it confirms the absence of brainstem death. This fact alone justifies special attention to this important reflex.

There is a need for consensus by physicians on what features of the PLR are clinically useful. Some authors rely on the percent reflex [1] while others use constriction velocity [2] or latency of constriction [5]. In judging the quality of the PLR, it may be unwise to place emphasis on a single parameter of the PLR waveform [6]. A proprietary algorithm designed to gauge the strength of the PLR, the Neurological Pupil Index (NPi), is scaled from 0 to 5, with 0 being an absent reflex [7]. The rarely observed “tonic light reflex” [8, 9] has a delayed constriction velocity and low reflex amplitude, and would require either several measurements or a longer light stimulus to determine the presence or absence of a PLR.

If the PRL is absent or severely depressed, then clinicians are required to draw upon their knowledge of the physiology and pharmacology of the pupil. A host of syndromes and drugs can depress or obliterate the PLR [9, 10]. The crossed afferent PLR pathway passes through the optic nerve, into the pretectal area, and then into the preganglionic pupilloctonstrictor (Edinger-Westphal) nucleus. This nucleus is embedded within the mesencephalic reticular formation, the integrity of which is necessary for the state of awareness. The efferent pathway of the PLR is important because of its tortuous pathway through the base of the pons, next to the tentorium, into the cavernous sinus, and then into the orbit. Each portion of the efferent pathway is vulnerable to compression by aneurysms, tumors, blood clots, and swollen brain tissue.

The clinician thus has several pathological processes to think about when evaluating the depressed PLR. Asymmetric efferent lesions produce anisocoria that can be detected more precisely with infrared pupillometry [1]. Asymmetric afferent lesions produce a relative afferent pupillary defect (RAPD). Most clinicians can perform the swinging flashlight test to evaluate this defect [9], but it is likely that within a few years the portable infrared method will be able to quantify the extent of RAPD.

In addition to pupil size, PLR, and anisocoria, there are other pupillary measures that have been considered for use in critical care units. Pupillary Reflex Dilation (PRD) is the dilation of the pupil that follows an alerting stimulus such as a tetanic electrical stimulus [10]. PRD during propofol administration is depressed in a dose-related manner by opioids [10] and has been used to predict pain-related behaviors in unconscious patients during subsequent noxious procedures [11, 12]. In awake subjects the PLR has been used for the same purpose [13]. Another pupillary measure is derived from the spontaneous fluctuations in pupil size during exposure to ambient light (termed pupillary unrest in ambient light (PUAL)). These fluctuations are depressed by opioids and can serve as a measure of opioid effect in awake subjects [14], but PUAL has not been evaluated in the critical care setting. Neither PRD nor PUAL can be measured with the pen-light examination, and thus require the use of portable infrared pupillometers in order to obtain accurate measurements.

## Conclusion

Several authors have stated that therapy has been altered because of pupillary signs detected by portable infrared pupillometry, but whether these measurements improved the clinical outcome has never been evaluated. If there is value to patient outcome by learning about the pupil, then it seems logical to obtain accurate measurements, especially when sequential measurements might indicate an evolving pathologic process that would go undetected by other bedside examinations or with imaging studies. Proof of added value from the use of portable infrared pupillometry may be difficult to demonstrate. As an example, most clinicians recognize the value of the pulse oximeter, but it remains a technology that has never been shown to improve outcome [15]. In the end, the value of any precise measurement depends on the clinician’s ability to interpret it. Physicians will eventually have to decide for themselves if precise pupillometry can improve the care of patients with critical illness.

## Abbreviations

PLR, pupillary light reflex; PUAL, pupillary unrest in ambient light; RAPD, relative afferent pupillary defect.
